# Correction: HIV-related stigma and uptake of antiretroviral treatment among incarcerated individuals living with HIV/AIDS in South African correctional settings: A mixed methods analysis

**DOI:** 10.1371/journal.pone.0259616

**Published:** 2021-11-01

**Authors:** Lucy Chimoyi, Christopher J. Hoffmann, Harry Hausler, Pretty Ndini, Israel Rabothata, Danielle Daniels-Felix, Abraham J. Olivier, Katherine Fielding, Salome Charalambous, Candice M. Chetty-Makkan

An additional affiliation is missing for the sixth author. Danielle Daniels-Felix is also affiliated with School of Public Health, University of the Witwatersrand, Johannesburg, South Africa.
