# Correction: Correction: HIV-related stigma and uptake of antiretroviral treatment among incarcerated individuals living with HIV/AIDS in South African correctional settings: A mixed methods analysis

**DOI:** 10.1371/journal.pone.0316768

**Published:** 2024-12-30

**Authors:** The PLOS ONE Staff

## Notice of Republication

There was an error in the affiliation for author Danielle Daniels-Felix in the correction published on November 1, 2019. This correction notice has been republished on December 17, 2024, to rectify this error. The publisher apologizes for the error. Please download this notice again to view the correct version.

## Supporting information

S1 File Originally published, uncorrected notice. (PDF)

S2 File Republished, corrected notice. (PDF)
