# Supplementary material for: HIV-related stigma and uptake of antiretroviral treatment among incarcerated individuals living with HIV/AIDS in South African correctional settings: A mixed methods analysis
Source: PLoS One. 2021 Jul 30;16(7):e0254975. doi: 10.1371/journal.pone.0254975 (PMC8323907; doi:10.1371/journal.pone.0254975)
Supplement: S1 File — (PDF) [file pone.0254975.s002.pdf]

SS001: INMATE SURVEY AT SIX MONTHS

Instructions: Read Verbatim: *"We are interested in knowing about the health of people in prison/correctional facilities (**use as appropriate**). I am going to ask you some questions about your feelings, behaviors, knowledge of HIV and TB, and experiences with health services in prison/corrections (**use as appropriate**). Some of these questions are sensitive, but it is very important that we know the truth about your feelings, behaviors, knowledge and experiences with health services so that we can better understand what helps and what gets in the way of giving health care in prison/ corrections (**use as appropriate**). There are no right or wrong answers. Please be honest when answering these questions. All of your answers will be kept confidential. Confidential means we will not tell your answers to anyone outside the research group and we will not share any of your answers with correctional officers. You don't have to answer any question if you don't want to and you may stop the questionnaire at any time."*

1. Language in which this interview was conducted: .... ☐☐

South Africa:
11 = Setswana
12 = Sesotho
13 = Zulu
14 = Xhosa
17 = Pedi
18 = Afrikaans
20 = English
99 = Other, specify: ______________________________

Zambia:
20 = English
22 = Bemba
23 = Nyanja
99 = Other, specify: ______________________________

PRISON/CORRECTIONAL HEALTH SERVICES

2. While in corrections/prisons, which of the following do you think has been the most important source of information about health? .... ☐

1 = Corrections/Prisons Officers
2 = Ministry Healthcare Workers
3 = Corrections Health Care Workers

4 = NGO Workers, specify: ____
5 = Other Inmates
6 = Peer educators
8 = Don't know/Unknown

9 = Other, specify: ____

3. Have you ever tried to seek health care while in corrections/prison? ....*1=Yes, 0=No, 3=No answer* ☐

If No or No answer, go to question 8.

3a. Have you tried to seek health care while in corrections/prisons in the last 12 months? ....
....*1=Yes, 0=No, 3=No answer* ☐

4. Are you confident that you will receive health care every time you need it in corrections/prison? ....*1=Yes, 0=No, 3=No answer* ☐

5. Are you confident that you will receive good quality health care while in corrections/prison? ....*1=Yes, 0=No, 3=No answer* ☐

6. Do you feel that you benefit from health care in corrections/prison? ....*1=Yes, 0=No, 3=No answer* ☐

7. Do you think the nurses and doctors at the prison clinic treat inmates with dignity and respect most of the time? ....*1=Yes, 0=No, 3=No answer* ☐

STANDARD OF LIVING

8. In terms of your sleeping arrangements, how do you currently sleep? .... ☐
   1 = Seated
   2 = Without a mattress on the floor
   3 = Two on a bed/mattress
   4 = Three or more on a bed/mattress
   5 = On your own bed/mattress

   9 = Other, specify: ____

9. Are you currently in a single cell? ....*1=Yes, 0=No, 3=No answer* ☐

If No or No answer, go to question 10.

   9a. What is the reason? ____
   Go to question 12.

10. At what hour do you typically go into lock up?... *HH:MM* ☐☐:☐☐

11. At what hour do you typically come out of lock up? .... *HH:MM* ☐☐:☐☐

12. While in prison/corrections this time, how often have you received money, food or other things from visiting family or friends? .... ☐
   1 = One or more times a month
   2 = About twice a year
   3 = Once a year
   4 = Never

13. Inside corrections/prisons have you ever shared a toothbrush? ....*1=Yes, 0=No, 3=No answer* ☐

14. Inside the corrections/prisons have you ever had to ask someone for food? ....*1=Yes, 0=No, 3=No answer* ☐

QUESTIONS ABOUT HIV

15. Have you received ARVs for HIV treatment at any time during this current stay in prison? ....*1=Yes, 0=No* □

If Yes, go to question 17.

16. What were the main reasons you did not start ARVs/HIV treatment?

16a. ______________________________

16b. ______________________________

16c. ______________________________

16d. ______________________________

16e. ______________________________

Go to question 22.

17. If you did start taking ARVs, are you still taking your ARVs for treatment of HIV? ....*1=Yes, 0=No* □

If Yes, go to question 19.

18. If you started ARVs but are no-longer taking ARVs, what were the main reasons you stopped taking ARVs?

18a. ______________________________

18b. ______________________________

18c. ______________________________

18d. ______________________________

18e. ______________________________

Go to question 21.

19. If you started ARVs and are still taking ARVs, have you experienced a gap of seven days or more in your ARVs during this current incarceration? ....*1=Yes, 0=No,* □

If No, go to question 21.

20. If you are still taking ARVs but have experienced a gap in your ARVs during this incarceration, what were the main reasons for not taking your ARVs?

    20a. ______________________________

    20b. ______________________________

    20c. ______________________________

    20d. ______________________________

    20e. ______________________________

21. Has getting ARVs for HIV treatment in corrections/prison made you more confident about looking after your health?.......... *1=Yes, 0=No, 3=No answer* ☐

22. Do you think you will have challenges accessing ARVs in the community clinic/health centre after you leave corrections/prisons? .... *1=Yes, 0=No, 3=No answer* ☐

CURRENT/PRIOR HISTORY OF TB

23. Have you been on treatment for TB at any time in the last 12 months (while in corrections/prison)?.......... *1=Yes, 0=No, 3=No answer* ☐

If No or No answer, go to question 24.

    23a. Have you experienced any interruption in TB treatment in the past 12 months while in corrections/prison? .... *1=Yes, 0=No, 3=No answer* ☐

    If No or No answer, go to question 24.

    23b. Can you tell us why there was an interruption in your TB treatment while in corrections/prison? __________

    ____

24. Have you ever been verbally abused, or think you have been verbally abused, in corrections/prison because of having TB? .... *1=Yes, 0=No, 3=No answer, 4=Never had TB* ☐

If No, No answer, or Never had TB, go to question 25.

    24a. How many times have you been verbally abused, or think you have been verbally abused, because of having TB? .... ☐

    1 = Once
    2 = A few times
    3 = Often
    8 = No answer

25. Have you ever been physically assaulted, or think you have been physically assaulted, in corrections/prison because of having TB? .... *1=Yes, 0=No, 3=No answer, 4=Never had TB* ☐

If No, No answer, or Never had TB, go to question 26.

25a. How many times have you been physically assaulted, or think you have been physically assaulted, because of having TB? .... ☐

1 = Once
2 = A few times
3 = Often
8 = No answer

KNOWLEDGE & BELIEFS

Read Verbatim: *"Now I am going to ask you some questions about HIV. Based on your understanding of HIV, please answer the following questions 'Yes' or 'No'. Remember, there are no right or wrong answers. You don't have to answer any question that you don't want to."*

**Question**	**Yes**	**No**	**No Answer/ Don't know**
26. Can HIV be cured by Western medicines?	☐	☐	☐
27. Can HIV be cured by traditional or herbal medicines?	☐	☐	☐
28. Can a person be cured of HIV through their faith or belief in God alone?	☐	☐	☐
29. Can HIV be treated but NOT cured with Western medicines?	☐	☐	☐
30. Can HIV be treated but NOT cured with traditional or herbal medicines?	☐	☐	☐
31. Can a person look healthy and still have HIV?	☐	☐	☐
32. Is unprotected sex safe when one is taking antiretroviral drugs (ARVs)?	☐	☐	☐
33. Can people receiving ARVs still transmit HIV to other people through unprotected sex?	☐	☐	☐
34. Is it acceptable to stop ARVs after gaining weight?	☐	☐	☐
35. Can one stop taking ARVs after a few years?	☐	☐	☐
36. Is it acceptable to miss a few tablets of ARVs?	☐	☐	☐

37. Do you know anyone who you believe was ill or died because of HIV/AIDS? ....*1=Yes, 0=No, 3=No answer* ☐

38. Do you believe that the disease called HIV/AIDS actually exists? ....*1=Yes, 0=No, 3=No answer* ☐

DEPRESSION

Read verbatim: *"We will now ask you about some of the ways you may have felt or behaved. Please indicate how often you have felt this way during the past week by choosing the one response for each question that best fits how you felt or behaved."*

	**Never/Rarely** *(less than 1 day/wk)*	**Some/A little of the time** *(1-2 days/wk)*	**Occasionally/ Moderately** *(3-4 days/wk)*	**All of the time** *(5-7 days/wk)*	**No Answer**
39. I was bothered by things that usually don't bother me.	☐	☐	☐	☐	☐
40. I had trouble keeping my mind on what I was doing.	☐	☐	☐	☐	☐
41. I felt sad or depressed.	☐	☐	☐	☐	☐
42. I felt that everything I did was an effort.	☐	☐	☐	☐	☐
43. I felt hopeful about the future.	☐	☐	☐	☐	☐
44. I felt fearful.	☐	☐	☐	☐	☐
45. My sleep was restless.	☐	☐	☐	☐	☐
46. I was happy.	☐	☐	☐	☐	☐
47. I felt lonely.	☐	☐	☐	☐	☐
48. I could not "get going".	☐	☐	☐	☐	☐

STIGMA

Read verbatim: "*Now I will ask you questions about how you may have felt or behaved recently, and about your experiences in prison/ corrections (**use as appropriate**). After listening to and understanding the question, please answer 'No' or 'Yes' to the best of your ability. Remember, there are no right or wrong answers. You don't have to answer any question that you don't want to."*

49. Have you ever felt ashamed because you have been in corrections/prison? ....*1=Yes, 0=No, 3=No answer* ☐

50. Do you feel you have lost respect from others or standing in your community because you have been in corrections/prison? ....*1=Yes, 0=No, 3=No answer* ☐

51. Have you ever felt ashamed to access health care in corrections/prison? .... *1=Yes, 0=No, 3=No answer* ☐

52. Have you ever felt ashamed because of your HIV status?..*1=Yes, 0=No, 3=No answer* ☐

53. Have you ever been reluctant to access ARVs in corrections/prison? ....*1=Yes, 0=No, 3=No answer* ☐

54. Do you think you are more comfortable with your HIV status here in corrections/prisons than you would be in the community? ....*1=Yes, 0=No, 3=No answer* ☐

55. Do you feel you have lost respect or standing in corrections/prisons because of your HIV status?..*1=Yes, 0=No, 3=No answer* ☐

56. Do you believe it is possible to keep your HIV status a secret while in corrections/prison? ....*1=Yes, 0=No, 3=No answer* ☐

57. Do you believe it is important to keep your HIV status a secret while in corrections/prison? ....*1=Yes, 0=No, 3=No answer* ☐

Read verbatim: *"I am now going to read a list of people. Can you please tell me whether you have told them that you are HIV positive?"*

58. Spouse or Partner?..*1=Yes, 0=No, 3=No answer, 7=NA* ☐

59. Other family member? ....*1=Yes, 0=No, 3=No answer* ☐

60. Friends on the outside of the correctional facility? ....*1=Yes, 0=No , 3=No answer* ☐

61. Other inmate (this incarceration)? ....*1=Yes, 0=No , 3=No answer* ☐

62. Work colleagues? ....*1=Yes, 0=No , 3=No answer* ☐

63. Corrections officer/staff member? ....*1=Yes, 0=No , 3=No answer* ☐

64. Other person not mentioned above? ....*1=Yes, 0=No , 3=No answer* ☐

If No or No answer, go to question 65.

64a. Who else have you told? ____

65. Are you comfortable talking about your HIV status with other inmates? .... *1=Yes, 0=No, 3=No answer* ☐

If No or No answer, go to question 66.

65a. How comfortable do you feel talking about your HIV status with other inmates? ....☐

1 = Not comfortable at all
2 = Somewhat comfortable
3 = Very comfortable

SHORT ANSWER

Read verbatim: *"Now I will ask you about any challenges you may have experienced accessing ARVs in prison/ corrections. After listening to the questions, please answer to the best of your ability. Remember, there are no right or wrong answers. You don't have to answer any question that you don't want to."*

66. What would you say are the biggest challenges – if any – to accessing ARVs in corrections / prisons?

66a. ____

66b. ____

66c. ____

66d. ____

66e. ____

Read verbatim: *"Thank you so much for your time today. This concludes the questionnaire. If you have any questions at all about either this questionnaire or anything else relating to the study, please don't hesitate to speak to either myself or another member of the TasP study team."*

STOP, form is complete.
