# Supplementary material for: HIV-related stigma and uptake of antiretroviral treatment among incarcerated individuals living with HIV/AIDS in South African correctional settings: A mixed methods analysis
Source: PLoS One. 2021 Jul 30;16(7):e0254975. doi: 10.1371/journal.pone.0254975 (PMC8323907; doi:10.1371/journal.pone.0254975)
Supplement: S2 File — (PDF) [file pone.0254975.s003.pdf]

INMATE IN-DEPTH INTERVIEWS

Introductory Script (follows full verbal consenting process):

Please read verbatim: ["Hello. My name is ________________________. Thank you for agreeing to an interview today. I want to reassure you that the information you share is confidential. What you say will not be connected back to you.

We are interested to hear about your experiences with health services both before and since the start of the TasP project/ offering immediate ART to inmates (which began on ***[INSERT DATE]***). You do not have to answer questions if you do not want to.

I want to remind you that the information you share is confidential. What you say will not be connected back to you. While the information gathered during this interview will be combined with other interviews and shared with the Zambian Corrections Service / Department of Corrections ***[use as appropriate]*** and the Ministry/Department of Health, no-one will know who said it, when it was said or where it was said. There are no 'right' or 'wrong' answers. We are interested in what you think and your experiences. Feel free to ask me any questions if something is unclear.

Do you have any questions before we begin?"]

General questions regarding how implementing TasP affects health services (better general services / better ART / more regular testing for HIV/TB) from patient & inmate perspectives

Part 1 - Background information:

This is to establish rapport…

a) How old are you? / Where are you from?
b) Are you married? Have you got any children?
c) Could you tell us how long you have been in prisons/ corrections and how you came to be here?
d) Could you tell us whether you are HIV-infected, and if so for how long? ***[IF NO, SKIP TO 1f]***
e) If so, are you receiving ART currently and if so for how long?
f) Can you tell me what are the major factors affecting your health in prison/ corrections right now?

   [PROBES]
   - Overcrowding / infrastructure
   - Sanitation
   - Nutrition
   - Stigma
   - Healthcare
   - Depression / Psychological issues
   - Substance use
   - Problems with other inmates and/or officers
g) Do you think these same factors affect other inmates too?
   - Why / why not?

Part 2 - Previous Experiences with Prison/ Correctional Health Services

We are now going to talk about healthcare in the prison in general (not the TasP project)

a) In general, when you need medical care, what is the process you go through to access it?

b) Have you ever been offered the chance to test for TB while in prison?
   - ***PROBE***: [Please describe how testing occurred and if you were diagnosed/ treated?]
   - ***PROBE***: [If on TB treatment have you experienced any challenges in accessing care or taking medication while in prison/ corrections?]

c) Can you tell us about any opportunities you have had to ***test*** for HIV while in prison/ corrections?
   - ***PROBE***:
   - [Where did ***HIV testing*** take place and who was offering it?
   - Did you want to receive ***HIV testing***? (please explain)
   - Was there anyone who was particularly supportive when it came to getting testing? (please explain)
   - Was there anyone who was particularly troublesome when it came to getting testing (please explain)]
   - What happened after you received your ***HIV test*** results (e.g. more counseling / referred for treatment/ ARVs)

d) Can you tell us about your experience getting ***ARVs/ care for your HIV***?
   - ***PROBE***:
   - Challenges, if any, in taking your ***ARVs/ treatment***
   - Anyone who was particularly supportive for getting ***ARVs/ care for your HIV***? (please explain)
   - Anyone who was particularly troublesome when it came to getting ***ARVs/ care for your HIV***? (please explain)

e) To your knowledge, have you ever had any other illness (not TB or HIV) while in prison/ corrections?

f) Overall, do you feel you are able to access health services here in prison / corrections in a timely manner? Please explain.

g) What do you feel are the positive features of health care in this prison / corrections facility – and what are the most challenging or negative features?
   - ***PROBE***: Do the challenges that you just mentioned influence your desire for care / ability to get care? (if so – how)

h) Do you think that the healthcare you receive in prison/ corrections is comparable to the healthcare you receive in the community? (Please explain why/why not)

i) Generally speaking, what are the things you would like to improve about ***health services*** in prison/ corrections?

Part 3 - Experiences with Healthcare under TasP Project (how do inmates perceive TASP project – b/c in Zambia new clinic / extra health workers; ZA may be more integrated into routine services).

I would now like to ask some questions about the TasP Project

a) Can you describe in your own words what you think the TasP project has been doing in this prison/ correctional facility?

b) Has the project/availability of immediate ART changed the way you personally access healthcare in prison/ corrections?
   - ***Please Describe***

c) Has there been any changes to the way health information or health services have been delivered in prisons/corrections since TasP/ immediate ART began (in the past XX months) that have changed the way you look after your own health?

d) From your own experience, can you describe the positive and negative aspects of these health services changes

[PROBES]

- Perceived timeliness / efficiency of services & treatment initiation
- Availability of health workers
- Attitudes of health workers, inmates, and corrections officers
- Access to information
- Acceptability of HTC in prison/ corrections
- HIV treatment/ ARVs in prison/ corrections
- Acceptability of testing for TB in prison/ corrections
- Immediate ART initiation
- Adherence support for ARVs and TB treatment

e) ***[For HIV-positive only]*** What, if any, issues have you experienced that prevented you from accessing or taking ARVs?

[PROBE]

- Stigma
- Attitudes
- Violence from other inmates/ corrections officers/ health workers in relation to HIV or TB status
- Other issues

<u>Part 4 - Future and Other issues</u>

a) ***[For TB and HIV-positive only]*** How do you feel you will manage your treatment in the long term in prison/ corrections?

b) ***[For TB and HIV-positive only]*** How do you feel you will manage your treatment once you are released to the community?

c) Is there anything else you would like to share before we finish?

[PROBE]

- Unmentioned health issues
