# Supplementary material for: Correction: Correction: HIV-related stigma and uptake of antiretroviral treatment among incarcerated individuals living with HIV/AIDS in South African correctional settings: A mixed methods analysis
Source: PLoS One. 2024 Dec 30;19(12):e0316768. doi: 10.1371/journal.pone.0316768 (PMC11684683; doi:10.1371/journal.pone.0316768)
Supplement: S1 File — (PDF) [file pone.0316768.s001.pdf]

PLOS ONE

CORRECTION

Correction: HIV-related stigma and uptake of antiretroviral treatment among incarcerated individuals living with HIV/AIDS in South African correctional settings: A mixed methods analysis

Lucy Chimoyi, Christopher J. Hoffmann, Harry Hausler, Pretty Ndini, Israel Rabothata, Danielle Daniels-Felix, Abraham J. Olivier, Katherine Fielding, Salome Charalambous, Candice M. Chetty-Makkan

An additional affiliation is missing for the sixth author. Danielle Daniels-Felix is also affiliated with School of Public Health, University of the Witwatersrand, Johannesburg, South Africa.

Reference

1. Chimoyi L, Hoffmann CJ, Hausler H, Ndini P, Rabothata I, Daniels-Felix D, et al. (2021) HIV-related stigma and uptake of antiretroviral treatment among incarcerated individuals living with HIV/AIDS in South African correctional settings: A mixed methods analysis. PLoS ONE 16(7): e0254975. https://doi.org/10.1371/journal.pone.0254975 PMID: 34329311

Check for updates

OPEN ACCESS

Citation: Chimoyi L, Hoffmann CJ, Hausler H, Ndini P, Rabothata I, Daniels-Felix D, et al. (2021) Correction: HIV-related stigma and uptake of antiretroviral treatment among incarcerated individuals living with HIV/AIDS in South African correctional settings: A mixed methods analysis. PLoS ONE 16(11): e0259616. https://doi.org/10.1371/journal.pone.0259616

Published: November 1, 2021

Copyright: © 2021 Chimoyi et al. This is an open access article distributed under the terms of the Creative Commons Attribution License, which permits unrestricted use, distribution, and reproduction in any medium, provided the original author and source are credited.
